# Placental Abruption, Preterm Delivery, and Cardiovascular Disease in Twin Offspring

**DOI:** 10.1161/JAHA.125.042202

**Published:** 2026-03-10

**Authors:** Rachel Lee, Emily B. Rosenfeld, Linda Valeri, William J. Kostis, Cande V. Ananth

**Affiliations:** ^1^ Division of Epidemiology and Biostatistics, Department of Obstetrics, Gynecology, and Reproductive Sciences Rutgers Robert Wood Johnson Medical School New Brunswick NJ; ^2^ Division of Maternal‐Fetal Medicine, Department of Obstetrics, Gynecology, and Reproductive Sciences Rutgers Robert Wood Johnson Medical School New Brunswick NJ; ^3^ Department of Biostatistics, Joseph L. Mailman School of Public Health Columbia University New York NY; ^4^ Department of Epidemiology Harvard T.H. Chan School of Public Health Boston MA; ^5^ Cardiovascular Institute of New Jersey Rutgers Robert Wood Johnson Medical School New Brunswick NJ; ^6^ Department of Medicine Rutgers Robert Wood Johnson Medical School New Brunswick NJ; ^7^ Department of Biostatistics and Epidemiology Rutgers School of Public Health Piscataway NJ; ^8^ Environmental and Occupational Health Sciences Institute Rutgers Robert Wood Johnson Medical School Piscataway NJ

**Keywords:** cardiovascular disease, causal mediation, placental abruption, preterm delivery, twins, Cardiovascular Disease, Congenital Heart Disease, Epidemiology, Pediatrics

## Abstract

**Background:**

In utero exposures to certain pathologies during pregnancy increase offspring cardiovascular disease (CVD) risks, and pregnancy complications and CVD risks are higher in twins than in singletons. However, the association between placental abruption and nonfatal incident CVD in twin offspring, and whether the abruption effects are mediated through preterm delivery, is unknown.

**Methods:**

We used the PACER (Placental Abruption and Cardiovascular Event Risk) offspring cohort, which links births to hospitalizations and mortality records in New Jersey from 1993 to 2020 (28 years of follow‐up) and is restricted to twin offspring. In a Cox model, we estimated the counterfactual decomposition of the abruption–CVD association into a natural direct and indirect effect through preterm delivery.

**Results:**

Of 116 796 twin births, 2597 (2.2%) were complicated by abruption. The rate of incident CVD was higher among abruption than nonabruption births (1560 versus 577 per 100 000 person‐years). Abruption was associated with a 2.47‐fold increased risk of CVD in the twin offspring, with similar risks for heart disease and stroke. Results indicated that 40% of the total effect was mediated through preterm delivery <37 weeks; the proportion mediated increased with earlier gestational ages at delivery. These risks persisted after accounting for unmeasured confounding bias.

**Conclusions:**

Placental abruption is associated with higher CVD risk in twins through early adulthood, with substantial mediation of effects through preterm delivery. Efforts to optimize obstetric care for pregnant women carrying twins to reduce the risk of abruption may be worthy of consideration. This may translate to public health benefits in reducing CVD in twin offspring.

## Graphical abstract

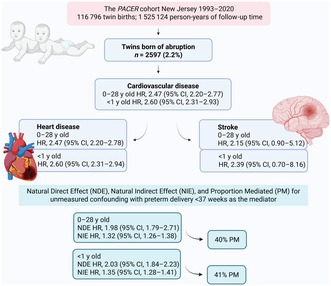

**Nonstandard Abbreviations and Acronyms**
NDE: natural direct effect
NIE: natural indirect effect
PACER: Placental Abruption and Cardiovascular Disease Event Risk

**Clinical Perspective**

**What Is New?**

- This study shows that twin offspring born of pregnancies complicated by abruption have a higher risk of immediate and long‐term cardiovascular disease than offspring born of pregnancies not complicated by abruption. Preterm delivery serves as an important pathway through which abruption confers higher risks of cardiovascular disease events in twin offspring. Preterm delivery’s role in these associations appears stronger with earlier gestational ages at delivery.

**What Are the Clinical Implications?**

- It is essential for physicians to screen for cardiovascular disease in twins born with adverse pregnancy outcomes, particularly placental abruption and preterm delivery. When counseling pregnant patients, it is crucial to discuss potential cardiovascular disease risks in twin offspring, which may translate to public health benefits.

Cardiovascular disease (CVD) is a leading cause of mortality in the United States,^1^ and CVD among young adults has been increasing.^2^ Obesity, smoking, substance use, physical activity, diet, hypertension, diabetes, and hyperlipidemia result from behavioral, socioeconomic, genetic, and environmental factors that contribute to CVD.^1^ The prevalence of obesity, diabetes, and hypertension, and the proportion of ischemic stroke and acute myocardial infarction in young patients have been increasing.^3^, ^4^, ^5^, ^6^ Findings from the CARDIA (Coronary Artery Risk Development in Young Adults) cohort have shown that young adulthood exposure to elevated blood pressure is associated with atherosclerosis in middle age.^7^, ^8^ In addition, high systolic and diastolic blood pressures and low‐density lipoprotein cholesterol increase the risks of coronary heart disease and heart failure later in life.^9^

In recent years, in utero exposures to certain pathologies, including pregestational and gestational diabetes,^10^ chronic hypertension, preeclampsia, eclampsia,^11^ fetal growth restriction,^12^ preterm delivery,^13^ and maternal obesity,^14^, ^15^ have been shown to affect vascular health in the offspring. These risk factors are associated with endothelial cell dysfunction and increased oxidative stress, potentially leading to insulin resistance, type 2 diabetes, hypertension, and atherosclerosis in the offspring.^16^ Meta‐analyses have shown that children and young adults born of preeclamptic pregnancies have higher systolic and diastolic blood pressures and body mass index,^17^, ^18^ which increases the risk of CVD events.

Placental abruption, the premature placental detachment, complicates about 1.2% to 2.5% of twin pregnancies, twice that of singleton deliveries.^19^, ^20^ An association between abruption and offspring CVD risks is plausible on 2 fronts. First, ischemia and inflammation are hallmarks of both conditions,^20^, ^21^ suggesting shared causes and similar pathophysiological mechanisms. Second, both conditions also share overlapping epidemiologic risk factors, including preexisting hypertension, preeclampsia, diabetes, obesity, and, notably, smoking and cocaine use.^21^ Preterm delivery itself has been associated with higher long‐term CVD risks in the offspring,^13^ and the risk of CVD morbidity is about 40% higher in twins than in singletons due to the dangers of being born earlier.^22^ Moreover, over two‐thirds of twin pregnancies complicated by abruption are delivered at preterm gestations; in contrast, ∼55% of singletons complicated by abruption are born preterm.^23^ Offspring CVD risks have been observed in singletons for those born of abruption compared with those without abruption,^24^ but, to date, no studies have examined the associations in twin offspring. Because abruption, preterm delivery, and CVD risks are substantially higher in twins than in singletons, we restricted this study to only twin offspring due to potential differences in physiological pathways that may influence the association between abruption and long‐term CVD.

We hypothesize that abruption—even in the absence of preterm delivery—will be associated with an increased burden of immediate and long‐term offspring CVD risks, and these risks will be substantially higher at preterm gestations. Through a causal decomposition (mediation) analysis, we sought to test this hypothesis to provide clues to unravel the complex relationships between placental abruption and offspring CVD risks among twins and how preterm delivery shapes these associations.

## METHODS

### Placental Abruption and Cardiovascular Event Risk Cohort

This study uses the PACER (Placental Abruption and Cardiovascular Event Risk) offspring cohort, which consists of all live births and fetal deaths from birth certificate data linked to delivery hospitalization, subsequent hospitalizations, and mortality files in New Jersey from 1993 to 2020, with 28 years of follow‐up.^25^ The linkages resulted in about 3.1 million offspring, with a 92.4% linkage rate for maternal hospitalization delivery. The PACER project received ethics approval from the Institutional Review Boards of Rutgers Biomedical and Health Sciences, NJ, and Rowan University, NJ. Because this was a retrospective observational study, consent from the subjects was not ascertained. Further details of the PACER project and the linkage methodology have been previously described.^25^ Due to the signed data use agreement, we are unable to make the data available to the public.

### Placental Abruption Exposure

Abruption is defined as the premature separation of the placenta with vaginal bleeding and at least 1 of the following conditions: fetal distress, uterine tenderness, or uterine hypertonicity. Abruption was determined from a birth certificate or maternal hospitalization delivery record with corresponding *International Classification of Diseases, Ninth Revision* (*ICD‐9*; 1993–2015) and *Tenth Revision* (*ICD‐10*; 2016–2020) codes (Table 1).

**Table 1:** *ICD‐9* and *ICD‐10* Codes for Placental Abruption and Cardiovascular Event Risk, 1993 to 2020

	*ICD‐9* (1993–2015)	*ICD‐10* (2016–2020)
Exposure		
Placental abruption	641.2	O45
Index event		
Cardiovascular disease (any)	398.91, 402, 410–414, 425, 427, 428, 430–438, 440–449, 745–747	I09.81, I11, I20–I25, I42, I46–I50, I60–I70, Q20–Q26
Heart disease (any)	402, 410–414, 425, 427, 428, 440–449, 745–747	I11, I20–I25, I42, I46–I50, I70, Q20–Q26
Congenital heart disease	745–747	Q20–Q26
Ischemic heart disease	410–414	I20–I25
Atherosclerosis	440	I70
Acute myocardial infarction	410	I21, I22
Hypertensive heart disease	402	I11
Heart failure	428	I50
Cardiomyopathy	425	I42
Cardiac arrhythmias	427	I46–I49
Stroke (any)	430–438	I60–I69
Ischemic stroke	430–432	I60–I62, I69
Hemorrhagic stroke	433–437	I63, I65–I67
Obstetric and labor and delivery complications		
Prepregnancy diabetes	250, 648.0	E08–E13, O24.0, O24.1, O24.3, O24.8
Gestational diabetes	648.8	O24.4, O24.9
Chronic hypertension	642.00–642.04, 642.20–642.24	O10.0, O10.4, O10.9
Gestational hypertension	642.30–642.34	O13
Preeclampsia without severe features	642.40–642.44	O14.0, O14.9
Preeclampsia with severe features	642.50–642.54	O14.1, O14.2
Eclampsia	642.60–642.64	O15
Superimposed preeclampsia	642.70–642.74	O11
Unspecified hypertension	642.90–642.94	O16

*ICD‐9* indicates *International Classification of Diseases, Ninth Revision*; and *ICD‐10*, *International Classification of Diseases, Tenth Revision*.

### Cardiovascular Disease Outcomes

We examined incident CVD, heart disease, and stroke hospitalizations as the primary outcome. Heart disease included congenital heart disease, ischemic heart disease, atherosclerosis, acute myocardial infarction, hypertensive heart disease, heart failure, cardiomyopathy, and cardiac arrhythmias. Hemorrhagic and ischemic strokes were categorized as stroke. *ICD‐9* and *ICD‐10* codes are shown in Table 1. We also examined CVD hospitalizations across all ages up to 28 years old, those restricted to occurring within 1 year of age, and events among those ≥1 years old.

### Exclusions

This study was limited to twins born alive. We excluded missing abruption status, singletons, triplets, and higher‐order multiple gestations, as well as fetal deaths. There were no additional exclusions for this study.

### Confounders

The confounders included child’s sex (female or male), matched sex in the twin set (male or female, both male, and both female) as a proxy for chorionicity due to severe underreporting of monochorionic or dichorionic chorionicity, maternal age, mother’s race/ethnicity (non‐Hispanic White, non‐Hispanic Black, Hispanic, and other), parity (1, 2, and ≥3), mother’s education (<9, 9–12, 13–16, and ≥17 years of completed schooling), marital status (married or single), insurance (Medicare, Medicaid, private, self‐pay, and others), tobacco smoking status (nonsmoker or smoker), hypertensive disorders of pregnancy (chronic hypertension, gestational hypertension, mild preeclampsia, severe preeclampsia, superimposed preeclampsia, and eclampsia), diabetes (prepregnancy and gestational), and birth year. Maternal age was examined as a continuous variable and centered by subtracting the mean age for the twin cohort. The “other” race category included Indian (from North America, Central America, South America, Eskimo, and Aleut), China, Japan, Hawaii, Filipines, other Asian or Pacific Islander (from India, Pakistan, Bangladesh, Cambodia, Thailand), Korea, Samoa, Vietnam, and Gauthe Philippines, other Asian or Pacific Islander (from India, Pakistan, Bangladesh, Cambodia, Thailand), Korea, Samoa, Vietnam, and Guam.

### Missing Data

We imputed missing covariates by creating 25 imputed data sets based on fully conditional specification models and applied Rubin’s rule to combine analyses and calculate the pooled variance.^26^ Imputations were based on the assumption that missing data were “missing at random.” Details about the multiple imputation approach have been previously published.^25^

### Statistical Analysis

**Figure 1:**
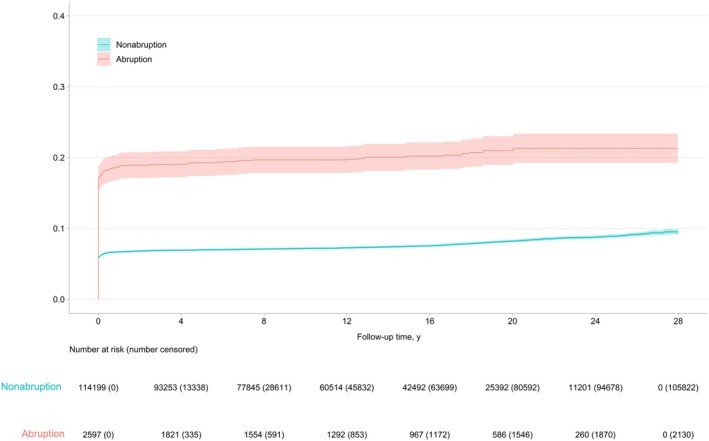
Kaplan–Meier curve showing the cumulative hazard of incident cardiovascular disease hospitalization among twin offspring born of pregnancies with and without placental abruption: Placental Abruption and Cardiovascular Event Risk, 1993 to 2020.

We determined the rate of incident CVD hospitalization per 100 000 person‐years of follow‐up. We fit Cox proportional hazards models to examine nonfatal incident CVD hospitalizations and estimated the hazard ratio (HR) and 95% CI after adjusting for the aforementioned confounders, accounting for twin cluster sets. We applied the inverse probability of sampling weights for unlinked records at birth and undertook a multiple imputation analysis for missing covariate data in all models. Missing covariate data were imputed 25 times based on the multivariate imputation by chained equations. All models were fit to each of the 25 multiply imputed data sets and subsequently combined based on Rubin’s principles.^27^ All deaths and no CVD hospitalizations by the end of the follow‐up period (December 31, 2020) were censored. The proportional hazards assumption was met as the cumulative risk in the Kaplan–Meier plot was parallel (Figure 1). Model diagnostics for assessing nonlinearity and examining influential observations were also met by plotting the Schoenfeld residuals and Martingale residuals for abruption and each covariate against the log follow‐up time (plots not shown). Statistical analyses were conducted in SAS (version 9.4; SAS Institute, Cary, NC).

We undertook a causal mediation analysis to decompose the natural direct effect (NDE), natural indirect effect (NIE), total effect, and proportion mediated of preterm delivery on abruption and nonfatal CVD association in the twin offspring. We examined the effects of preterm delivery at <37, <34, and <32 weeks and CVD hospitalization up to 28 years old and at <1 year old. Mediation effects were not estimable for CVD hospitalizations ≥1 year due to small numbers. We estimated the adjusted HR and 95% CI fitted by the Cox proportional hazards model with time to first CVD hospitalization as the time scale. The 95% CI was derived from 1000 bias‐corrected accelerated bootstrap resampling methods.

All statistical analyses were conducted in RStudio (version 4.3.2) using the R package *CMAVerse*.^28^

### Sensitivity Analysis

We undertook 3 sensitivity analyses. To address unmeasured confounding bias, we calculated the bias‐corrected HRs by dividing the observed HR and 95% CI by the joint bounding factor derived as (HR_EU_ × HR_UD_)/(HR_EU_ + HR_UD_ − 1).^29^ HR_EU_ and HR_ED_ refer to the hazard ratios of the exposure‐unmeasured confounder and the unmeasured confounder‐outcome scenarios. Next, the mediation decomposition rests on assumptions of no unmeasured confounding of the exposure–outcome, mediator–outcome, and exposure–mediator relations, and exposure that confounds the mediator–outcome relation^30^ and the positivity assumption.^31^, ^32^ To examine if the causal estimates of HRs were affected by unmeasured confounding bias, we undertook a bias analysis without assumptions on unmeasured confounding. We obtained the joint bounding bias factor for the NDE and NIE (on the HR scale) as $\left[1+\left(\gamma -1\right)\pi_{1}\right]/\left[1+\left(\gamma -1\right)\pi_{0}\right]$ and $\left[1+\left(\gamma -1\right)\pi_{0}\right]/\left[1+\left(\gamma -1\right)\pi_{1}\right]$, respectively.^33^ γ denotes the effect of a binary unmeasured confounder on the outcome. $\pi_{1}\;\text{and}\;\pi_{0}$ are the prevalence of the unmeasured confounder among those with and without abruption, respectively. The bias‐corrected HRs for the NDE and NIE were derived by dividing the HR_NDE_ and HR_NIE_ by the corresponding bias factors. The bias‐corrected total effect was calculated by multiplying the HRs of the bias‐corrected NDE and NIE.^33^

We examined whether maternal CVD drove the strong associations between abruption and twin offspring CVD risks, and we replicated the primary associations after excluding women with a CVD diagnosis. Finally, we adjusted for the confounding effects of assisted reproductive technology conception on the abruption–CVD outcomes in the offspring as a sensitivity analysis and not in the primary analysis, because abruption may feature on the causal pathway between assisted reproduction method and CVD.^34^

## RESULTS

Of 116 796 twin deliveries, 2597 (2.2%) had a recorded diagnosis of abruption. Abruption rates increased with earlier gestational ages and maternal tobacco smoking. Twins born of pregnancies complicated by abruption had lower 5‐minute Apgar scores and higher rates of fetal distress, respiratory distress syndrome, and newborn seizures. Although twin‐to‐twin transfusion syndrome was rare (0.4%), rates of abruption were higher for offspring born with this condition. More than half (55.1%) of all twins were born preterm (<37 weeks); in contrast, 82.7% of all abruption twin births were born preterm (Table 2).

**Table 2:** Maternal and Offspring Characteristics and Perinatal Outcomes Based on Placental Abruption in Twin Births: Placental Abruption and Cardiovascular Event Risk, 1993 to 2020

	Total cohort number (%_col_)	Placental abruption		No placental abruption number (%_col_)
		Number (%_col_)	(%_row_)	
Total twin deliveries	116 796 (100.0)	2597 (100.0)	(2.2)	114 199 (100.0)
Maternal characteristics				
Year of birth				
1993–1995	9238 (7.9)	224 (8.6)	(2.4)	9014 (7.9)
1996–2000	19 406 (16.6)	512 (19.7)	(2.6)	18 894 (16.5)
2001–2005	23 805 (20.4)	561 (21.6)	(2.4)	23 244 (20.4)
2006–2010	24 958 (21.4)	558 (21.5)	(2.2)	24 400 (21.4)
2011–2015	22 760 (19.5)	404 (15.6)	(1.8)	22 356 (19.6)
2016–2020	16 629 (14.2)	338 (13.0)	(2.0)	16 291 (14.3)
Gestational age at birth, wk				
20–27	4575 (3.9)	504 (19.4)	(11.0)	4071 (3.6)
28–31	7055 (6.0)	559 (21.5)	(7.9)	6496 (5.7)
32–33	10 173 (8.7)	370 (14.2)	(3.6)	9803 (8.6)
34–36	42 584 (36.5)	716 (27.6)	(1.7)	41 868 (36.7)
37–38	41 569 (35.6)	387 (14.9)	(0.9)	41 182 (36.1)
39–40	6863 (5.9)	48 (1.8)	(0.7)	6815 (6.0)
41	163 (0.1)	0 (0.0)	(0.0)	163 (0.1)
42–44	22 (0.0)	0 (0.0)	(0.0)	22 (0.0)
Unknown	3792 (3.2)	13 (0.5)	(0.3)	3779 (3.3)
Preterm birth, wk				
≥37	48 617 (41.6)	435 (16.8)	(0.9)	48 182 (42.2)
<37	64 387 (55.1)	2149 (82.7)	(3.3)	62 238 (54.5)
<34	21 803 (18.7)	1433 (55.2)	(6.6)	20 370 (17.8)
<32	11 630 (10.0)	1063 (40.9)	(9.1)	10 567 (9.3)
Unknown	3792 (3.2)	13 (0.5)	(0.3)	3779 (3.3)
Maternal age, y				
15–19	2702 (2.3)	58 (2.2)	(2.1)	2644 (2.3)
20–24	10 971 (9.4)	240 (9.2)	(2.2)	10 731 (9.4)
25–29	23 899 (20.5)	531 (20.4)	(2.2)	23 368 (20.5)
30–34	42 618 (36.5)	862 (33.2)	(2.0)	41 756 (36.6)
35–39	27 767 (23.8)	692 (26.6)	(2.5)	27 075 (23.7)
40–44	6899 (5.9)	178 (6.9)	(2.6)	6721 (5.9)
45–49	1666 (1.4)	28 (1.1)	(1.7)	1638 (1.4)
Unknown	274 (0.2)	8 (0.3)	(2.9)	266 (0.2)
Parity				
Parity 1	32 997 (28.3)	731 (28.1)	(2.2)	32 266 (28.3)
Parity 2	43 423 (37.2)	958 (36.9)	(2.2)	42 465 (37.2)
Parity ≥3	38 106 (32.6)	897 (34.5)	(2.4)	37 209 (32.6)
Unknown	2270 (1.9)	11 (0.4)	(0.5)	2259 (2.0)
Maternal race or ethnicity				
Non‐Hispanic White	64 269 (55.0)	1501 (57.8)	(2.3)	62 768 (55.0)
Non‐Hispanic Black	16 206 (13.9)	444 (17.1)	(2.7)	15 762 (13.8)
Hispanic	17 972 (15.4)	372 (14.3)	(2.1)	17 600 (15.4)
Other	16 166 (13.8)	275 (10.6)	(1.7)	15 891 (13.9)
Unknown	2183 (1.9)	5 (0.2)	(0.2)	2178 (1.9)
Maternal education, y				
0–8	2305 (2.0)	50 (1.9)	(2.2)	2255 (2.0)
9–12	32 035 (27.4)	776 (29.9)	(2.4)	31 259 (27.4)
13–16	52 919 (45.3)	1266 (48.7)	(2.4)	51 653 (45.2)
≥17	22 994 (19.7)	459 (17.7)	(2.0)	22 535 (19.7)
Unknown	6543 (5.6)	46 (1.8)	(0.7)	6497 (5.7)
Marital status				
Single	25 444 (21.8)	608 (23.4)	(2.4)	24 836 (21.7)
Married	87 571 (75.0)	1987 (76.5)	(2.3)	85 584 (74.9)
Unknown	3781 (3.2)	2 (0.1)	(0.1)	3779 (3.3)
Maternal tobacco smoking				
Nonsmoker	104 141 (89.2)	2377 (91.5)	(2.3)	101 764 (89.1)
Smoker	7502 (6.4)	220 (8.5)	(2.9)	7282 (6.4)
Unknown	5153 (4.4)	0 (0.0)	(0.0)	5153 (4.5)
Hypertensive disorders				
Normotensive	85 272 (73.0)	2098 (80.8)	(2.5)	83 174 (72.8)
Chronic hypertension	1748 (1.5)	63 (2.4)	(3.6)	1685 (1.5)
Gestational hypertension	6543 (5.6)	100 (3.9)	(1.5)	6443 (5.6)
Preeclampsia, no severe features	7010 (6.0)	133 (5.1)	(1.9)	6877 (6.0)
Preeclampsia with severe features	4695 (4.0)	107 (4.1)	(2.3)	4588 (4.0)
Superimposed preeclampsia	397 (0.3)	5 (0.2)	(1.3)	392 (0.3)
Eclampsia	1135 (1.0)	29 (1.1)	(2.6)	1106 (1.0)
Unknown	9996 (8.6)	62 (2.4)	(0.6)	9934 (8.7)
Diabetes				
No diabetes	105 769 (90.6)	2399 (92.4)	(2.3)	103 370 (90.5)
Prepregnancy diabetes	2093 (1.8)	49 (1.9)	(2.3)	2044 (1.8)
Gestational diabetes	6580 (5.6)	149 (5.7)	(2.3)	6431 (5.6)
Chorioamnionitis				
Absent	106 969 (91.6)	2442 (94.0)	(2.3)	104 527 (91.5)
Present	2330 (2.0)	132 (5.1)	(5.7)	2198 (1.9)
Unknown	7497 (6.4)	23 (0.9)	(0.3)	7474 (6.5)
Primary insurance status				
Medicare	337 (0.3)	6 (0.2)	(1.8)	331 (0.3)
Medicaid	11 722 (10.0)	303 (11.7)	(2.6)	11 419 (10.0)
Private	81 536 (69.8)	1927 (74.2)	(2.4)	79 609 (69.7)
Self‐pay	3285 (2.8)	91 (3.5)	(2.8)	3194 (2.8)
Others	6402 (5.5)	140 (5.4)	(2.2)	6262 (5.5)
Unknown	13 514 (11.6)	130 (5.0)	(1.0)	13 384 (11.7)
Offspring characteristics				
Child sex				
Female	58 222 (49.8)	1277 (49.2)	(2.2)	56 945 (49.9)
Male	58 567 (50.1)	1320 (50.8)	(2.3)	57 247 (50.1)
Unknown	7 (0.0)	0 (0.0)	(0.0)	7 (0.0)
Matched twins sex				
Male and female	42 777 (36.6)	983 (37.9)	(2.3)	41 794 (36.6)
Both male	37 169 (31.8)	835 (32.2)	(2.3)	36 334 (31.8)
Both female	36 850 (31.6)	799 (30.0)	(2.1)	36 071 (31.6)
5‐min Apgar score				
0–3	1388 (1.2)	114 (4.4)	(8.2)	1274 (1.1)
4–7	5353 (4.6)	443 (17.1)	(8.3)	4910 (4.3)
8–10	104 663 (89.6)	2032 (78.2)	(1.9)	102 631 (89.9)
Unknown	5392 (4.6)	8 (0.3)	(0.1)	5384 (4.7)
Breech presentation				
Absent	92 073 (78.8)	2060 (79.3)	(2.2)	90 013 (78.8)
Present	23 847 (20.4)	523 (20.1)	(2.2)	23 324 (20.4)
Unknown	876 (0.8)	14 (0.5)	(1.6)	862 (0.8)
Fetal distress				
Absent	111 686 (95.6)	2359 (90.8)	(2.1)	109 327 (95.7)
Present	4234 (3.6)	224 (8.6)	(5.3)	4010 (3.5)
Unknown	876 (0.8)	14 (0.5)	(1.6)	862 (0.8)
Respiratory distress syndrome				
Absent	114 118 (97.7)	2458 (94.6)	(2.2)	111 660 (97.8)
Present	1435 (1.2)	120 (4.6)	(8.4)	1315 (1.2)
Unknown	1243 (1.1)	19 (0.7)	(1.5)	1224 (1.1)
Newborn seizure				
Absent	116 382 (99.6)	2590 (99.7)	(2.2)	113 792 (99.6)
Present	46 (0.0)	2 (0.1)	(4.3)	44 (0.0)
Unknown	368 (0.3)	5 (0.2)	(1.4)	363 (0.3)
Twin–twin transfusion syndrome				
Absent	108 838 (93.2)	2559 (98.5)	(2.4)	106 279 (93.1)
Present	461 (0.4)	15 (0.6)	(3.3)	446 (0.4)
Unknown	7497 (6.4)	23 (0.9)	(0.3)	7474 (6.5)
Age at first cardiovascular disease hospitalization, y				
No hospitalizations	107 952 (92.4)	2130 (82.0)	(2.0)	105 822 (92.7)
At birth	6831 (5.8)	396 (15.2)	(5.8)	6435 (5.6)
After birth to 4	1273 (1.1)	49 (1.9)	(3.8)	1224 (1.1)
5–9	160 (0.1)	7 (0.3)	(4.4)	153 (0.1)
10–14	175 (0.1)	6 (0.2)	(3.4)	169 (0.1)
15–19	254 (0.2)	7 (0.3)	(2.8)	247 (0.2)
20–24	124 (0.1)	2 (0.1)	(1.6)	122 (0.1)
25–28	27 (0.0)	0 (0.0)	(0.0)	27 (0.0)

The rate of incident CVD hospitalization was higher among twins born of abruption than without abruption (1560 versus 577 per 100 000 person‐years; median [interquartile range], 13.3 [4.9–19.9] and 13.0 [6.5–19.3] years) (Table 3). Heart disease and stroke hospitalization rates were also higher in twins born of pregnancies complicated by abruption than without abruption. Heart disease hospitalizations consisted mostly of congenital heart disease, and the majority of the events occurred within the first year of birth. Heart disease rates remained elevated for twins born of abruption than without abruption, even beyond 1 year of birth, with higher rates of cardiac arrhythmias (60 per 100 000 person‐years) than congenital heart disease (34 per 100 000 person‐years).

**Table 3:** Rates of Incident Cardiovascular Morbidity in Twin Offspring Born of Pregnancies With and Without Placental Abruption: Placental Abruption and Cardiovascular Event Risk, 1993 to 2020

Cardiovascular disease	No placental abruption (*n* = 144 199)			Placental abruption (*n* = 2597)		
	Total person‐years	Person‐years median (IQR)	CVD events number (rate per 100 000 person‐years)	Total person‐years	Person‐years median (IQR)	CVD events number (rate per 100 000 person‐years)
All ages (0–28 y)						
Any cardiovascular disease	1 450 887	13.0 (6.5–19.3)	8377 (577)	29 930	13.3 (4.5–19.9)	467 (1560)
Heart disease	1 449 889	13.0 (6.5–19.4)	8211 (566)	29 909	13.4 (4.5–19.9)	459 (1535)
Congenital heart disease	1 441 734	13.1 (6.6–19.4)	6920 (480)	29 699	13.5 (4.7–20.0)	412 (1387)
Cardiac arrythmias	1 443 562	13.1 (6.6–19.4)	1263 (87)	29 720	13.5 (4.8–20.0)	57 (192)
Stroke	1 440 776	13.7 (7.8–19.8)	177 (12)	29 625	15.4 (9.0–21.0)	8 (27)
Age <1 y						
Any cardiovascular disease	103 812	1.0 (1.0, 1.0)	7382 (7111)	1998	1.0 (1.0, 1.0)	435 (21772)
Heart disease	103 794	1.0 (1.0, 1.0)	7312 (7045)	1997	1.0 (1.0, 1.0)	431 (21582)
Congenital heart disease	103 728	1.0 (1.0, 1.0)	6680 (6440)	1994	1.0 (1.0, 1.0)	402 (20160)
Cardiac arrythmias	103 672	1.0 (1.0, 1.0)	668 (644)	1993	1.0 (1.0, 1.0)	39 (1957)
Stroke	103 626	1.0 (1.0, 1.0)	78 (75)	2321	1.0 (1.0, 1.0)	…*
Age ≥1 y						
Any cardiovascular disease	1 450 683	13.7 (7.8–19.8)	995 (69)	29 922	15.4 (9.0–21.0)	32 (107)
Heart disease	1 449 702	13.7 (7.8–19.8)	899 (62)	29 902	15.4 (9.0–21.0)	28 (94)
Congenital heart disease	1 441 613	13.7 (7.8–19.8)	240 (17)	29 694	15.4 (9.0–21.0)	10 (34)
Cardiac arrythmias	1 447 023	13.7 (7.8–19.8)	595 (41)	29 810	15.4 (9.0–21.0)	18 (60)
Stroke	1 440 758	13.7 (7.8–19.8)	99 (7)	29 624	15.4 (9.0–21.0)	…*

* Number suppressed for <5 events. CVD indicates cardiovascular disease; and IQR, interquartile range.

**Figure 2:**
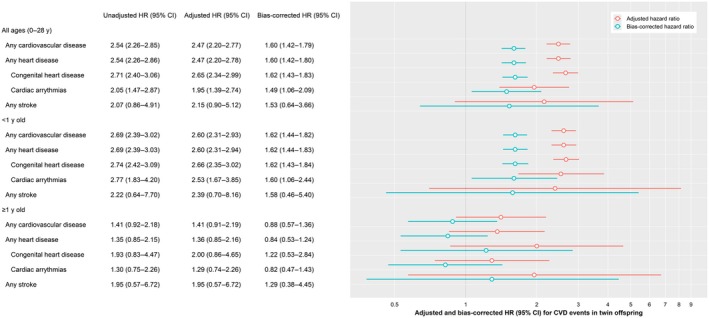
Risks of CVD events in twin offspring born of pregnancies complicated by placental abruption: Placental Abruption and Cardiovascular Event Risk, 1993 to 2020. The unadjusted HR (95% CI) is not shown in the forest plot. CVD indicates cardiovascular disease; and HR, hazard ratio.

The Kaplan–Meier curve showed higher cumulative risk in births complicated by abruption compared with nonabruption across all ages starting from birth (Figure 1). Twin offspring born of abruption had higher CVD risk, with slightly higher risks for heart disease (HR, 2.47 [95% CI, 2.20–2.78]) than stroke (HR, 2.15 [95% CI, 0.90–5.12]) (Figure 2). These CVD risks associated with abruption were similar (2.60‐fold higher) at <1 year old. Even after the first year of life, there was a 1.41‐fold higher risk of CVD for twin offspring born of abruption.

**Figure 3:**
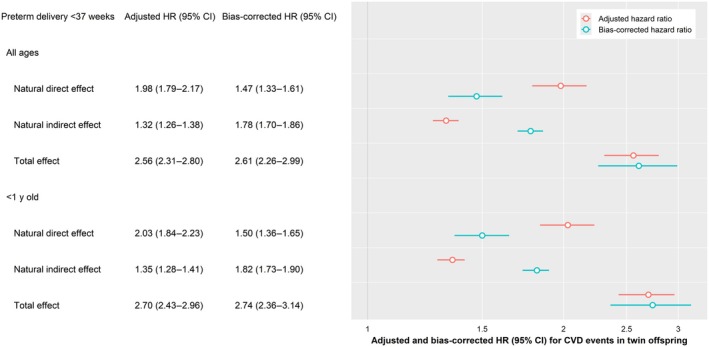
Natural direct and natural indirect effects mediated by preterm delivery (<37 wk) of the association between placental abruption and CVD events among twin offspring associated with placental abruption: Placental Abruption and Cardiovascular Event Risk, 1993 to 2020. CVD indicates cardiovascular disease; and HR, hazard ratio.

After adjusting for confounders, the HR for the abruption‐CVD association in twin offspring was higher for the effect independent of preterm delivery (HR, 1.98 [95% CI, 1.79–2.17]) than the effect mediated through preterm delivery (HR, 1.32 [95% CI, 1.26–1.38]). Correspondingly, the proportion mediated through preterm delivery at <37 weeks was 40% (Table 4 and Figure 3). As the gestational age at delivery decreased, the mediated effects became stronger, suggesting a higher proportion mediated through preterm delivery. Mediation effects were similar for CVD hospitalizations up to 28 years old and at <1 year old.

**Table 4:** Hazard Ratio (95% CI) of the Natural Direct and Natural Indirect Effects Mediated Through Preterm Delivery of the Association Between Placental Abruption and Cardiovascular Disease Among Twin Births: Placental Abruption and Cardiovascular Event Risk, 1993 to 2020

	Hazard ratio (95% CI)					
	All ages (0–28 y)			<1 y old		
	Unadjusted	Adjusted	Bias corrected*	Unadjusted	Adjusted	Bias corrected*
Preterm delivery <37 wk						
Natural direct effect	2.03 (1.82–2.19)	1.98 (1.79–2.17)	1.47 (1.33–1.61)	2.06 (1.84–2.27)	2.03 (1.84–2.23)	1.50 (1.36–1.65)
Natural indirect effect	1.31 (1.25–1.37)	1.32 (1.26–1.38)	1.78 (1.70–1.86)	1.34 (1.28–1.40)	1.35 (1.28–1.41)	1.82 (1.73–1.90)
Total effect	2.61 (2.34–2.80)	2.56 (2.31–2.80)	2.61 (2.26–2.99)	2.77 (2.50–3.01)	2.70 (2.43–2.96)	2.74 (2.36–3.14)
Proportion mediated (%)	38	40	71	40	41	71
Preterm delivery <34 wk						
Natural direct effect	1.38 (1.26–1.51)	1.35 (1.22–1.48)	1.00 (0.90–1.10)	1.39 (1.25–1.53)	1.35 (1.22–1.49)	1.00 (0.90–1.10)
Natural indirect effect	1.92 (1.77–2.10)	1.92 (1.75–2.09)	2.59 (2.36–2.82)	2.08 (1.93–2.29)	2.09 (1.91–2.28)	2.82 (2.58–3.08)
Total effect	2.67 (2.40–2.92)	2.61 (2.35–2.87)	2.59 (2.14–3.09)	2.78 (2.50–3.08)	2.74 (2.46–3.01)	2.82 (2.33–3.40)
Proportion mediated (%)	77	78	…†	81	82	…†
Preterm delivery <32 wk						
Natural direct effect	1.26 (1.16–1.37)	1.24 (1.11–1.35)	0.92 (0.82–1.00)	1.36 (1.15–1.39)	1.23 (1.11–1.35)	0.91 (0.82–1.00)
Natural indirect effect	2.02–(1.85–2.21)	2.02 (1.83–2.22)	2.73 (2.47–3.00)	2.14 (1.90–2.37)	2.14 (1.92–2.34)	2.89 (2.59–3.16)
Total effect	2.71 (2.49–2.96)	2.66 (2.39–2.93)	2.50 (2.03–3.00)	2.85 (2.60–3.15)	2.77 (2.49–3.06)	2.63 (2.13–3.16)
Proportion mediated (%)	80	81	…†	82	83	…†

Hazard ratios were adjusted for the confounding effects of the child’s sex, matched sex, mother’s race, age, parity, education, marital status, insurance, tobacco smoking status, hypertensive disorders of pregnancy, prepregnancy and gestational diabetes, and birth year. Missing data were imputed 25 times, and the hazard ratio and 95% CI were derived from the pooled variance from 25 imputations.

* Bias‐corrected HR for unmeasured confounding (see the Methods section for details). Bias‐corrected proportion mediated=HR_NDE_(HR_NIE_−1)/(HR_NDE_×HR_NIE_−1).

^†^ Proportion mediated is not estimable because the NDE and NIE are in the opposite direction of the null. Mediation analysis for ≥1 y old are not shown due to small number of cardiovascular disease events. HR indicates hazard ratio; NDE, natural direct effect; and NIE, natural indirect effect.

### Sensitivity Analysis

CVD risks persisted and were slightly lower after accounting for unmeasured confounding for the overall abruption and CVD association (Figure 2). Bias‐corrected NDE decreased, and NIE increased, therefore increasing the mediation effects through preterm delivery after accounting for unmeasured confounding (Table 4 and Figure 3). Associations between abruption and CVD hospitalizations were similar after excluding women with a CVD diagnosis (Table 5). The associations remained unchanged after adjustments for assisted reproductive technology conception (Table 5).

**Table 5:** Sensitivity Analyses for Cardiovascular Disease Risk in the Twin Offspring Born of Pregnancies Complicated by Placental Abruption: Placental Abruption and Cardiovascular Event Risk, 1993 to 2020

	Hazard ratio (95% CI)					
	All ages (0–28 y)			<1 y old		
	Unadjusted*	Adjusted†	Adjusted‡	Unadjusted*	Adjusted†	Adjusted‡
Cardiovascular disease (any)	2.55 (2.28–2.87)	2.48 (2.21–2.79)	2.46 (2.19–2.77)	2.70 (2.40–3.04)	2.62 (2.33–2.95)	2.60 (2.31–2.93)
Heart disease (any)	2.56 (2.28–2.88)	2.49 (2.21–2.80)	2.46 (2.19–2.77)	2.71 (2.40–3.05)	2.62 (2.32–2.96)	2.60 (2.30–2.93)
Congenital heart disease	2.73 (2.42–3.08)	2.66 (2.36–3.01)	2.64 (2.33–2.98)	2.75 (2.44–3.12)	2.68 (2.37–3.04)	2.66 (2.35–3.01)
Cardiac arrythmias	1.99 (1.42–2.79)	1.89 (1.35–2.66)	1.96 (1.39–2.74)	2.64 (1.73–4.03)	2.41 (1.57–3.70)	2.55 (1.68–3.88)
Stroke (any)	2.16 (0.90–5.19)	2.21 (0.93–5.27)	2.17 (0.91–5.17)	2.35 (0.67–8.21)	2.46 (0.72–8.39)	2.40 (0.71–8.10)

The inverse probability of sampling weights was incorporated in the Cox proportional hazards regression models to account for unlinked records. Missing data were imputed 25 times, and the hazard ratio and 95% CI were derived from pooled variance from 25 imputations and accounting for twin clusters. CVD indicates cardiovascular disease.

* Excluding offspring with a maternal history of CVD.

^†^ Excluding offspring with maternal history of CVD, and hazard ratios were adjusted for confounding effects of the child’s sex, matched sex, race, mother’s age, parity, mother’s education, marital status, insurance, tobacco smoking status, hypertensive disorders of pregnancy, prepregnancy and gestational diabetes, and birth year.

^‡^ Hazard ratios were adjusted for the confounding effects of the child’s sex, matched sex, race, mother’s age, parity, mother’s education, marital status, insurance, tobacco smoking status, hypertensive disorders of pregnancy, prepregnancy and gestational diabetes, birth year, and assisted reproductive technologies.

## DISCUSSION

### Main Findings

Twin offspring born of pregnancies complicated with abruption have higher immediate and long‐term risks of CVD hospitalization compared with those born without abruption. Heart disease and stroke risks were higher during infancy, but elevated risks persisted beyond 1 year of age. Most heart disease hospitalizations were due to congenital heart disease and cardiac arrhythmias. Preterm delivery <37 weeks strongly mediated the association, with the mediated proportion becoming stronger with earlier gestational ages. The effect mediated through preterm delivery increased after bias‐correction for unmeasured confounding.

### Comparison to Other Studies

No study to date has examined the association between abruption and the risks of CVD among twin offspring. Only 1 study has examined the effect of abruption and offspring CVD risks in singletons. This study included all deliveries (n=217 910) from 1991 to 2014 with 18 years of follow‐up time in a tertiary medical center in Israel and found a slight increase in CVD events in offspring (HR, 1.12 [95% CI, 0.60–2.11]) for offspring born with abruption (n=1003). However, there were few CVD events in this cohort (n=10).^24^ The PACER cohort included longer follow‐up time, more births complicated with abruption (n=2597), more CVD events (n=467), and higher CVD risks in twins associated with abruption births. Because abruption, preterm delivery, CVD, and maternal CVD risk factors are all increased in twins compared with singletons, both abruption and CVD risk may be higher in twins than in singletons, although further studies are needed in singleton offspring. This study strengthens the growing literature on how abruption exposure shapes long‐term health in the offspring.

### Biological Pathways

Offspring born with placental abruption share similar causes and associations with those born with fetal growth restriction and preeclampsia due to placental ischemia or uteroplacental insufficiency,^35^ endothelial cell dysfunction,^36^ and oxidative stress.^37^ There are 2 pathways through which abruption can occur: chronic and acute. Abnormal placentation results in chronic abruption, and premature placental separation leads to acute placental separation. In chronic abruption, early abnormal spiral artery development leads to decidual necrosis, placental inflammation and possible infarction, and ultimately low‐pressure venous hemorrhage resulting in a small area of placental separation and fetal growth restriction in the twins^21^ associated with cerebral vasodilation, suggesting chronic hypoxemia.^38^ This may be a possible pathway by which CVD is increased in twin offspring born of chronic abruption. In acute severe abruption, high‐pressure arterial hemorrhage in the central area of the placenta extensively dissects through the placental–decidual interface, causing complete or nearly complete placental separation. A small portion of cases are caused by sudden mechanical events or by rapid uterine decompression after the first twin is delivered, or rapid decompression of polyhydramnios.^21^ As a result, placental abruption could affect metabolic pathways through fetal asphyxia, hypoxia, or ischemia as blood flow and the delivery of oxygen and nutrients are diminished in partial abruptions or entirely ceased in a complete abruption.^30^ Perinatal asphyxia can result in neurologic insult; respiratory distress; pulmonary hypertension, hepatic, myocardial, and renal dysfunction; and risk factors for future CVD, depending on the severity and timing of hypoxic insult.^39^ Uterine crowding, overdistention, and unequal distribution of shared placental mass specific to twin births may contribute to fetal growth restriction^40^ and lead to cardiac remodeling and metabolic changes. The compounding effects of stress initiated by abruption in twins and shared space in the uterine environment may explain the worsening cardiovascular outcomes in twins than in singletons.

People born preterm have a higher risk of ischemic heart disease and long‐term CVD.^13^, ^41^ Cardiac structural and functional alterations may explain these increased risks. Adverse modifications in left and right ventricular structure and function worsen with blood pressure elevation, impaired myocardial functional reserve, and increased diffuse myocardial fibrosis, which may lead to impaired left ventricular diastolic function.^42^ In our study, ∼60% of the increased CVD risk associated with abruption remains unexplained by preterm delivery (∼30% after bias‐correction for unmeasured confounders). This suggests preterm delivery alone is not driving the increased CVD risk in twins but that abruption itself confers CVD risk. Because abruption is unpredictable, and interventions to prolong preterm gestations independent of abruption have been met with little success, efforts to identify other pathways independent of preterm delivery through which chronic and acute abruption affects CVD events in twin offspring may be worthy of consideration. Previous studies have shown that there is an increased risk of maternal CVD after a twin pregnancy.^43^ It may be that this higher demand on the cardiovascular system with twins leads to an increase in an inflammatory response that may affect the cardiovascular function of the offspring. A placental abruption may exacerbate these changes, leading to worse outcomes in the offspring.

Finally, maternal risk factors for abruption, such as preexisting hypertension, obesity, and smoking during pregnancy, could affect the offspring’s CVD health. Maternal hypercholesterolemia and obesity could induce changes in the fetal aorta, leading to early atherosclerotic lesions in children born with normal lipid profiles.^44^ Smoking during pregnancy has been shown to increase fetal arterial resistance and higher blood pressure in children at 2 to 3 years old, suggesting cardiovascular consequences in the early postnatal years.^45^, ^46^

### Clinical Implications

Since the 1980s, the twinning rate has increased by 33%, primarily thought to be driven by the rise in assisted reproductive technology,^47^ which is estimated to contribute to 12.1% of all twin births.^48^ Multifetal gestations carry increased risks of abruption and preterm delivery as compared with singleton pregnancies.^49^, ^50^, ^51^ For those pregnant with twins, tobacco and cocaine cessation is recommended to decrease their risk of abruption.^20^ Given that our understanding of long‐term childhood outcomes is evolving, there are no current societal recommendations for tailored follow‐up or management of these children. However, in those who have an abruption episode, the pediatrician should monitor for CVD events during childhood and likely throughout the life course.

### Strengths and Limitations

Using a population‐based cohort, this is the most extensive study to assess abruption and immediate and long‐term CVD risks in twin offspring in the United States. We were able to adjust for multiple confounders due to the linkage between the birth certificate and hospitalization data at the time of birth. We used inverse probability of sampling weights and imputations for missing data and accounting for the clustering of twins in a pregnancy.

Further studies are needed to understand the effects of medications to treat maternal complications during pregnancy on offspring’s CVD health. Paternal comorbidities at the time the offspring were conceived were not available. Although this may present a limitation in the data set, the bias‐corrected HR would account for such unmeasured confounding effects of maternal medication and paternal comorbidities. We did not consider the time‐dependent confounders in the mediation analysis. This might violate the assumption that no unmeasured confounding exists on the exposure that confounds the mediator–outcome relation. There may be measurement errors in diagnosing abruption due to the accuracy of birth certificates and hospitalization data. This may result in minimal selection bias because the prevalence of abruption in twins is similar to what is reported in the literature.^20^ We could not determine the type of twinning, such as monochorionic or dichorionic gestation, on the abruption and CVD association due to >75% missing chorionicity data. The effects of chorionicity on how abruption may shape CVD risks in twin offspring need to be further evaluated because monochorionic twins have a higher incidence of preterm delivery, morbidities, and congenital heart defects compared with dichorionic twins.^52^, ^53^, ^54^ Despite the 28 years of follow‐up, the absolute CVD rates are likely lower, and longer follow‐up time is needed to study CVD risks in early and late adulthood. Our cohort captures hospitalization events only in New Jersey, so we could not identify events that could occur in other states or account for loss of follow‐up due to migration. This study was limited to CVD events, and further research needs to be conducted on CVD mortality risks.

## CONCLUSIONS

Twin offspring born of pregnancies complicated by abruption have increased risks of CVD observed as early as <1 year old and that persist into early young adulthood. Although preterm delivery mediates about two‐fifths of the increased CVD risk, abruption could impact CVD risks through metabolic programming and cardiac remodeling due to stressors in utero. There is a growing focus in public health and medicine to shift the focus from intervention of CVD events to preventive medicine in the womb to optimize offspring CVD health before early developmental stages.

## Sources of Funding

The PACER project is supported by the National Heart, Lung, and Blood Institute (R01‐HL150065), National Institutes of Health, awarded to Drs Ananth and Kostis. Dr Ananth is additionally supported by the National Institute of Environmental Health Sciences (R01‐ES033190), National Institutes of Health.

## Disclosures

None.
